# Effects of developmental exposure to silver in ionic and nanoparticle form: A study in rats

**DOI:** 10.1186/s40199-016-0162-9

**Published:** 2016-10-06

**Authors:** Mohammad Charehsaz, Karin Sørig Hougaard, Hande Sipahi, Asiye Işın Doğan Ekici, Çiğdem Kaspar, Mustafa Culha, Ülkü Ündeğer Bucurgat, Ahmet Aydin

**Affiliations:** 1 Department of Toxicology, Yeditepe University, Faculty of Pharmacy, Atasehir Istanbul, Turkey; 2 National Research Center for the Working Environment, Danish Nanosafety Centre, Copenhagen, Denmark; 3 Department of Pathology, Yeditepe University, Faculty of Medicine, Istanbul, Turkey; 4 Department of Biostatistics, Yeditepe University, Faculty of Medicine, Istanbul, Turkey; 5 Department of Genetics and Bioengineering, Yeditepe University, Istanbul, Turkey; 6 Department of Toxicology, Hacettepe University, Faculty of Pharmacy, Ankara, Turkey

**Keywords:** Silver, Oxidative stress, Hippocampal sclerosis, Pregnancy, Rat

## Abstract

**Background:**

Evaluations of silver in both nanoparticle (Ag-NPs) and ionic forms indicate some adverse effects on living organisms, but little is known about their potential for developmental toxicity. In this study, developmental toxicity of Ag-NPs (from 0.2 to 20 mg/kg/day) and ionic Ag (AgNO_3_, 20 mg Ag/kg/day) were investigated in rats.

**Methods:**

Animals were dosed by gavage from gestation day 7 − 20. The day after parturition, dams and pups were sacrificed and Ag level assessed in several maternal and pup organs. In addition, hepatotoxicity and oxidative stress parameters and histopathology were evaluated.

**Results:**

No treatment related effects were found for gestational parameters including pregnancy length, maternal weight gain, implantations, birth weight and litter size at any dose level of Ag-NPs. Maternal weight gain was lower in dams receiving AgNO_3_ compared to the other groups, suggesting that the ionic form may exert a higher degree of toxicity compared to the NP form. Tissue contents of Ag were higher in all treated groups compared to control dams and pups, indicating transfer of Ag across the placenta. The findings furthermore suggest that Ag may induce oxidative stress in dams and their offspring, although significant induction was only observed after dosing with AgNO_3_. Histopathological examination of brain tissue revealed a high incidence of hippocampal sclerosis in dams treated with nanoparticle as well as ionic Ag.

**Conclusion:**

The difference in offspring deposition patterns between ionic and NP Ag and the observations in dam brain tissue, requires scrutiny, and, if corroborated, indicate that ionic and NP forms maybe need separate risk assessments and that the hazard ratings of silver in both ionic and nanoparticle forms should be increased, respectively.

**Trial registration:**

Not applicable.

**Graphical abstract:**

Developmental Toxicity of Ag-NPs. 
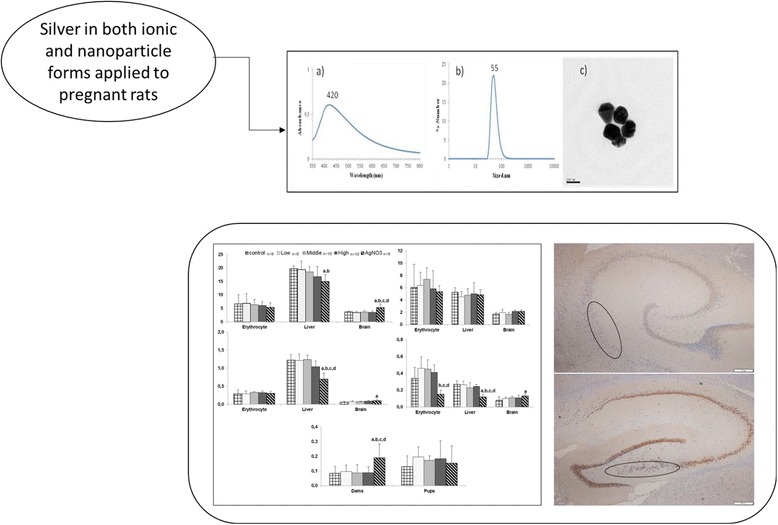

## Background

Silver has remarkably strong antimicrobial and antifungal properties. This attribute is a major reason for the wide use of silver nanoparticles (Ag-NPs) in personal care, household and medical products, as well as in textiles and food industry. Commercial products that contain Ag-NPs are one of the most rapidly growing classes of products. Roughly 24 % of the products currently registered in nanoproduct databases claim to contain Ag-NPs and its use is foreseen to increase in the future [1].

Some studies on the toxicity of Ag-NPs indicate that they cause oxidative stress, which subsequently drives inflammatory, cytotoxic and genotoxic responses [2]. Thus, Ag^+^ from Ag-NPs seems to distribute easily to maternal tissues and has been associated with oxidative stress in the adult organism [3, 4]. In the adult, Ag administered in both ionic and nanoparticle forms can distribute to the brain [5–7]. The Ag-NPs have been described to increase production of reactive oxygen species (ROS) in the hippocampus and cause neuronal cell damage [8]. Similarly, Rahman et al. reported that Ag-NPs may induce neurotoxicity by generation of oxidative stress and alteration of gene expression [9]. Also silver in ionic form strongly increases the production of reactive oxygen species, decreases intracellular reduced glutathione levels and increases the susceptibility of human skin fibroblasts to H_2_O_2_-induced cell death [10]. Hadrup et al. suggest that ionic Ag and Ag-NPs have similar neurotoxic effects, as the 2 forms of Ag in their 28 day study caused similar changes in brain dopamine levels [11]. The similarity in effect may be due to the release of ionic Ag from the surface of Ag-NPs. Although the health implications of silver in nanoparticle and ionic forms may differ to some degree, the overall concepts that guide assessment of those risks would in many ways be expected to be similar.

Hong et al. conducted a repeated-dose oral toxicity study of Ag-NPs on reproduction and development in rats. In the maternal rat, Ag levels were increased in lung, liver and kidney [12]. The distribution of Ag from mother to the fetus was not investigated in that study. Melnik et al. reported that Ag in nanoparticle form transfer from the mother to her offspring through the placenta and breast milk in rats [13]. Austin et al. examined the distribution of Ag in pregnant mice and embryos/fetuses following intravenous injections of Ag-NPs or ionic silver in mice. Authors reported that Ag from Ag-NPs did not cross the placental barrier in large amounts, but did accumulate in visceral yolk sac and maternal tissues [14]. The distribution of Ag to tissues in the offspring following maternal administration of ionic silver has not been assessed apart from one study including distribution to only the fetal brain, where Ag was shown to accumulate [15]. Further investigation is needed to reach a more complete understanding whether the tissue distribution differs with administration of Ag-NPs and ionic silver.

Yu et al. investigated the potential effects of orally administered Ag-NPs on pregnancy and embryo-fetal development in rats. In the dams, a significant decrease in catalase and glutathione reductase activities, as well as a reduction in glutathione content was observed in liver tissue. No treatment related changes in maternal, classical gestational and litter endpoints was observed, even at very high dose levels [3]. Another study investigated oral administration of Ag-NPs on reproduction and development in mice. There were no obvious signs of maternal toxicity at any dose level, but the number of non-viable fetuses was significantly increased in dams exposed to a single oral dose of 10 mg/kg Ag-NPs, but not higher doses. The authors argued that this finding might be due to facilitation of aggregates at higher gut concentrations, preventing internalization via the gastrointestinal tract and thereby reducing fetotoxicity at higher doses [16]. The potential negative effects of Ag NPs on development and reproduction have yet to be fully clarified [17]. In comparison, only one rodent study has investigated the developmental toxicity of orally administered ionic silver (190 mg kg/day) on gestational day 1–20. Post-implantation lethality was increased and fetal body mass reduced compared to the control group. 2.5 % of the investigated embryos had visible abnormalities and postnatal lethality was massive [18].

To our knowledge, the toxicity and distribution of Ag in ionic and nanoparticle forms have never been compared previously in relation to gestational and developmental parameters. We therefore aimed to compare the developmental effects of silver in ionic and nanoparticle form, by administering Ag ions as AgNO_3_ or citrate-reduced Ag-NPs by gavage to pregnant Sprague Dawley rats and investigate: 1) Ag distribution to several maternal and fetal organs 2) developmental toxicity (litter parameters, body weight etc.), and 3) and several indices of maternal toxicity, namely oxidative stress parameters, hepatotoxicity and histopathology.

## Methods

### Synthesis and characterization of Ag-NPs

Citrate-capped Ag-NPs were synthesized by chemical reduction according to Lee and Meisel [19]. Briefly, a 90 mg of AgNO_3_ (≥99.5 % purity) (Sigma, USA) was dissolved in a 500 ml of deionized water (Simplicity UV, France) and heated. Then, a 10 mL of 1 % trisodium citrate solution (Merck, Germany) was added drop-wise to the boiling solution under vigorous mixing in darkness. Change of color to slightly yellow indicated reduction of silver ions to Ag-NPs.

The mechanism of reaction could be expressed as follows:

$$ 4{\mathrm{Ag}}^{+} + {\mathrm{C}}_6{\mathrm{H}}_5{\mathrm{O}}_7{\mathrm{Na}}_3 + 2{\mathrm{H}}_2\mathrm{O}\ \to\ 4{\mathrm{Ag}}^0 + {\mathrm{C}}_6{\mathrm{H}}_5{\mathrm{O}}_7{\mathrm{H}}_3 + 3{\mathrm{Na}}^{+} + {\mathrm{H}}^{+} + {\mathrm{O}}_2 $$

To prevent aggregation, Ag-NP suspensions were synthesized freshly thrice a week. The concentration of total Ag in Ag-NPs suspensions and AgNO_3_ solutions was monitored by atomic absorption spectrometry (AAS) (Analytic Jena, Zeenit 700, Germany) every morning before administration. Size distribution and zeta potential of the Ag-NPs were assessed by Dynamic Light Scattering (DLS) at 25 °C (Malvan, Zetasizer NanoZS, UK). The Ag-NPs were further characterized by UV-Vis spectroscopy (Thermo Evolution 300, USA) and visualized by TEM (Jeol, 2100 HR, operating at 200 Kv, USA). AgNO_3_ solution was produced freshly thrice a week using the same materials as in the synthesis of nanoparticles.

### Animals and treatment

Male and female Sprague-Dawley rats were obtained from the Yeditepe University Medical School Experimental Research Center (YUDETAM) and housed at controlled room temperature (21 ± 1 °C) with a 12:12 h light-dark cycle in polypropylene cages with bedding. The experimental protocol was approved by the Ethic Committee of Yeditepe University. For mating, two mature females were placed overnight in a cage with a mature male. Mating was confirmed by the presence of sperm in vaginal smear, designated day 0 of gestation (GD0), and the pregnant dam were weighed and moved to pair wise housing with another pregnant female. Dams were assigned to 5 groups of 10 animals each and gavaged once daily from GD7 to GD20 with 0 (deionized water), low (0.2 mg/kg), middle (2 mg/kg) or high (20 mg/kg) dose Ag-NPs suspension or with 20 mg Ag/kg as AgNO_3_. Dosing volume was 4 ml/kg. Dams were weighed daily and dosing adjusted accordingly. The oral route was selected due to the extensive use of Ag in the water treatment, toothpaste, reusable bottles and, kitchen utensils (Park et al. [20]) (Vance et al. [1]). From GD18, dams were housed separately and monitored daily for birth. The day of spontaneous delivery was designated as postnatal day (PND) 1. On PND2, pups were counted and sexed. Dams and individual pups were weighed and sacrificed by decapitation. Trunk blood was collected from dams and all pups in a litter, the latter pooled to a single litter sample (BD Vacutainer, UK), and then centrifuged for 10 min at 4500 rpm at 4 °C. After separation of plasma, the buffy coat was removed and the packed cells were washed thrice with two volumes of isotonic saline. Then, a known volume of erythrocytes was lysed with cold distilled water (1:4), stored at 4 °C for 15 min and the cell debris was removed by centrifugation (3200 rpm at 4 °C for 10 min). Several organs were dissected from dams (brain, heart, lungs, liver, kidneys, spleen, ovaries, and uterus) and 6–7 pups/litter (brain, lungs, liver, kidneys and stomachs with milk), weighed and frozen on dry ice. All samples were stored at -80 °C until analysis. For histopathological examination, brain (coronal sections from temporal and hippocampal areas), lung, spleen, heart, kidney, uterus, ovaries and liver from 5 dams/group, and brain, heart, liver, lung and kidney from one pup in the same litters were fixed in 10 % formalin (J.T.Baker, Netherlands).

### Determination of Ag in tissue and milk

Tissue samples from all dams and 2–3 pups/litter were digested with concentrated nitric acid (Sigma, USA) using a microwave digestion system (CEM Mars 5, USA). The concentration of Ag was then determined by graphite furnace atomic absorption spectrometer (AAS ZEEnit 700) with Zeeman background correction according to the manufacturer's instructions.

### Biochemical and inflammatory analysis

Commercially available enzyme-linked immunosorbent assay (ELISA) kits were used for determination of ALT (Uscn, USA), AST and IL-6 (SunRed, China) levels in plasma by microplate spectrophotometer (Multiskan Ascent, Finland) using the manufacturer’s instruction.

### Measurement of oxidative stress parameters

Brain and liver samples from dams and pups (pooled from randomly selected 2–3 pups/litter) were minced and homogenized with a glass homogenizer in 1.15 % cold potassium chloride solution (Riedel-de Haen, Germany), and centrifuged at 4500 rpm and 4 °C for 15 min. Supernatants were analyzed for oxidative stress parameters as were the erythrocyte part of blood samples. For offspring, blood samples were pooled from all pups in each litter.

Protein content in erythrocyte lysate and tissue homogenates was determined spectrophotometrically according to the Lowry method [21]. Superoxide dismutase (SOD) activity was measured according to Aydin et al. [22] catalase (CAT) and glutathione peroxidase (GPx) according to Celep et al. [23] and malondialdehyde (MDA) by monitoring thiobarbituric acid according to Jamall and Smith [24]. Plasma NO_2_^-^/NO_3_^-^ levels were measured using the Griess reaction according to Tracey et al. [25].

### Histopathology

Following routine tissue processing, the tissues were embedded in paraffin. 4 μm thick sections from each paraffin block were stained with hematoxylin (Bio Optica, Italy) and eosin (DDK, Italy) for histopathological evaluation under digital light microscope (Olympus BX53, Japan). Micro photos were taken by Olympus DP 73 (Japan) micro camera.

Immunohistochemistry for Glial fibrillary acidic protein (GFAP) and neuron-specific nuclear protein (Neu-n): 2 sections (4 μm thick) from each paraffin block for the immunohistochemistry were deparaffinized in xylene and dehydrated in graded ethyl alcohol. Following deparaffinization, the slides were boiled for 20 min in 10 mM citrate buffer, pH 6.0, followed by cooling at room temperature for 20 min, then rinsed with distilled water. The slides were immersed for 30 min in 0.3 % hydrogen peroxide in methanol for endogenous peroxide inactivation followed by three washes in phosphate buffer saline (PBS, pH 7.4) at room temperature. Subsequently, non-specific binding was blocked by PBS containing 1 % goat serum and 1 % bovine serum albumin which was applied for 30 min. Next, GFAP (Glial Fibrillary acidic Protein Ab-6; 200 μg/ml, mouse monoclonal antibody, Lab Vision Corporation, Thermo Fisher Scientific, Fremont, CA) and Neu-N (prediluted anti-neuron specific nuclear protein; Clone A60, IHCR1001-6, Chemicon IHC Select research,human, mouse, rat monoclonal Antibody, EMD Millipore Corporation, Temecula, CA, USA) were applied for 30 min at room temperature, respectively. After washing in PBS, peroxidase activity was localized with chromogen 3,3’-diaminobenzidine (DAB; DAKO Liquid DAB-Substrate-chromogen K-3466, CA, USA) and 0.03 % hydrogen peroxide. Sections were counter-stained with Haematoxylen, cleaned and mounted. Negative control studies were performed concurrently in the absence of the primary antibody. Positive control studies were also performed simultaneously in sections of a human hippocampus section. Brown staining in cytoplasm of glial cells was considered as “positive” and no staining as “negative” for GFAP and brown staining in cytoplasm of neuronal cells was considered as “positive” and no staining as “negative” for Neu-n.

Histopathological examination was evaluated by a pathologist blind to the exposure status of the animals.

### Statistical analysis

All analyses were performed using SPSS software version 21.0. The litter was used as the unit of analysis. The Kolmogorov–Smirnov test was used to evaluate normal distribution. Homogeneity of variance was analyzed by Levene’s test. Data are presented as mean ± standard deviation (SD). When more than two groups were compared, data with normal distribution and homogeneity of variance were analyzed by ANOVA, data with normal distribution but heterogeneity of variance by Welch ANOVA. When overall analyses were statistically significant, pairwise post-hoc tests were performed using Tukey’s test (for data with normal distribution and homogeneity of variance) and Dunnett’s T3 test (for data with normal distribution and heterogeneity of variance). In case of non-normally distributed data, the Kruskal-Wallis test was applied, with Mann-Whitney U test for pairwise comparisons. Organ weights for dams and pups were expressed as a relative organ weights (percentage of body weight). A *p* < 0.05 was considered statistically significant.

## Results

### Characterization of Ag-NPs suspension

**Fig. 1:**
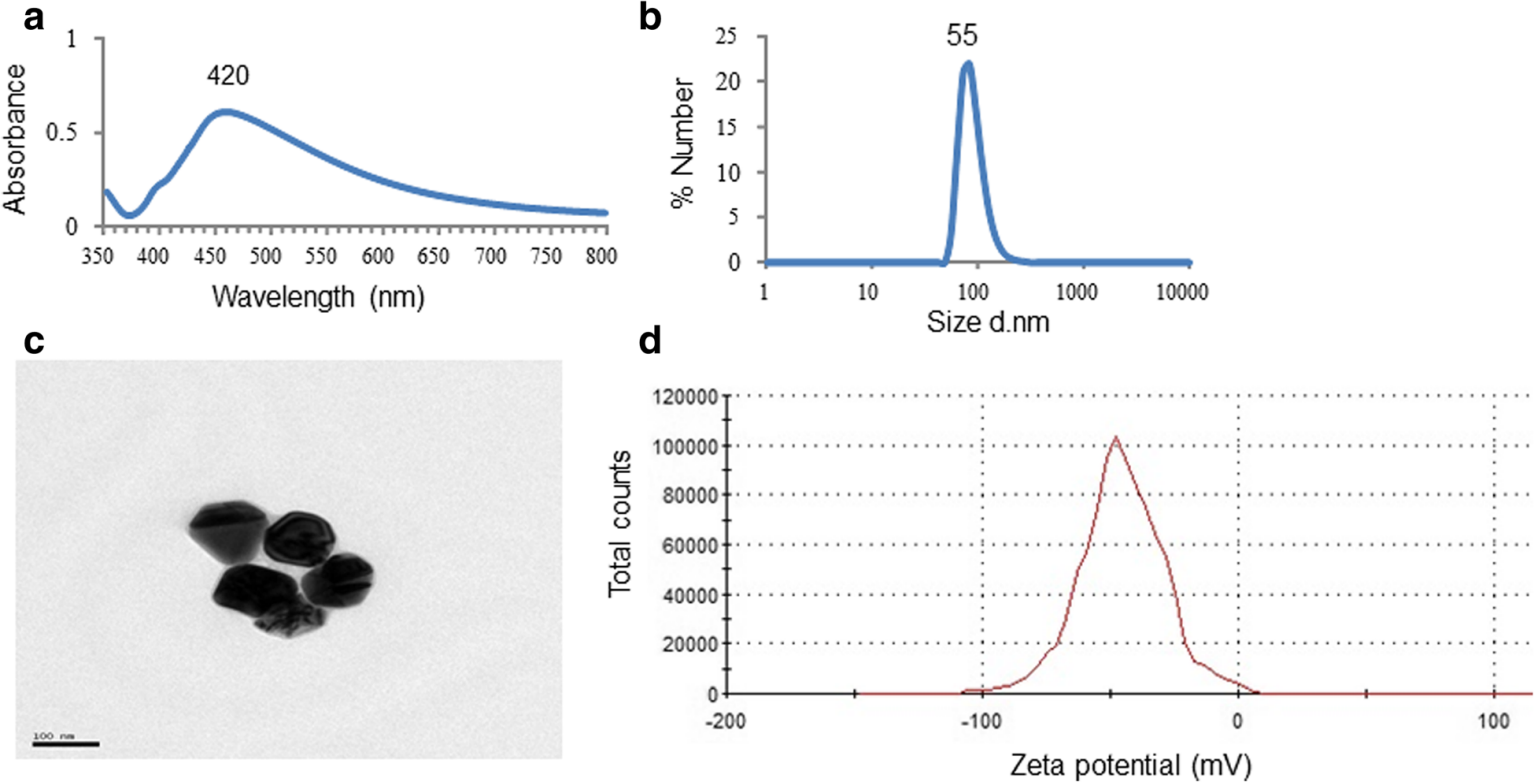
**a** UV/Vis absorption spectrum of Ag-NPs, **b** DLS spectrum of Ag-NPs colloidal suspension, **c** TEM image of Ag-NPs, **d** Zeta potential of synthesized Ag-NPs

DLS analysis revealed that the average hydrodynamic radius of Ag-NPs was 55 nm, which was confirmed by TEM (Fig. 1). A zeta potential of approximately -45 Mv was measured. The maximum absorption of the solution occurred at around 420 nm.

### Maternal and developmental observations

Maternal body weight gain from was significantly lower in dams receiving AgNO_3_ compared to low (*p* < 0.05) and high dose Ag-NPs (*p* < 0.001). Pregnancy length, number of implantations or fetal resorptions, litter size, sex distribution and offspring weights did not vary significantly between groups (Table 1).

**Table 1:** Pregnancy outcome and litter data

Dams and litters	Control	0.2 mg/kg Ag-NPs	2 mg/kg Ag-NPs	20 mg/kg Ag-NPs	20 mg/kg AgNO_3_
No. of dams (litters)	*n* = 10 (9)	*n* = 10 (8)	*n* = 10 (10)	*n* = 10 (10)	*n* = 10 (9)
Body weight gain (g), GD 7-20	77.1 ± 8.9	83.8 ± 9.5	80.6 ± 9.2	87.9 ± 7.8	71.9 ± 11.4^a,b^
Pregnancy length (days)	20.9 ± 0.8	21.0 ± 0.0	20.8 ± 0.4	21.1 ± 0.3	21.1 ± 0.8
No. of implantation sites	12.6 ± 1.7	13.0 ± 1.8	13.1 ± 1.7	14.6 ± 1.1	14.4 ± 2.3
No. of resorptions	1.7 ± 0.9	1.3 ± 1.6	0.9 ± 0.9	1.6 ± 1.0	1.0 ± 0.8
Litter size	10.9 ± 2.2	11.7 ± 2.5	12.2 ± 1.5	13.0 ± 1.5	13.0 ± 2.5
Offspring body weight (g)	6.7 ± 1.3	5.9 ± 0.7	5.7 ± 0.8	5.4 ± 0.5	6.0 ± 1.3
% males	51.1 ± 16.2	52.8 ± 9.4	49.6 ± 13.2	56.6 ± 5.3	53.4 ± 14.1

Results were expressed as the mean ± SD. ^a^Significantly different from low dose (*p* < 0.05), ^b^Significantly different from high dose (*p* < 0.05)

For dams, the relative weights of the heart, uterus and brain were significantly higher at the highest dose of Ag-NPs compared to the controls (*p* < 0.05) (Table 2).

**Table 2:** Relative organ weights (% of body weight) of dams and pups

Dams	Dose levels (mg/kg)				
	Control (*n* = 9)	0.2 Ag-NPs (*n* = 8)	2 Ag-NPs (*n* =10)	20 Ag-NPs (*n* = 10)	20 AgNO_3_ (*n* = 9)
Brain	0.87 ± 0.10	0.87 ± 0.09	0.93 ± 0.06	0.98 ± 0.06^a,b^	0.95 ± 0.05
Lung	0.46 ± 0.05	0.52 ± 0.09	0.58 ± 0.16	0.52 ± 0.06	0.61 ± 0.16
Heart	0.34 ± 0.02	0.36 ± 0.08	0.36 ± 0.03	0.39 ± 0.04^a^	0.38 ± 0.03
Liver	3.82 ± 0.27	3.97 ± 0.31	3.77 ± 0.33	3.78 ± 0.28	4.06 ± 0.30
Spleen	0.21 ± 0.03	0.20 ± 0.02	0.19 ± 0.02	0.21 ± 0.04	0.21 ± 0.03
Kidney (Right)	0.39 ± 0.04	0.38 ± 0.02	0.37 ± 0.04	0.41 ± 0.06	0.42 ± 0.03
Kidney (Left)	0.38 ± 0.04	0.37 ± 0.03	0.37 ± 0.03	0.41 ± 0.03	0.42 ± 0.04
Ovary (Right)	0.03 ± 0.01	0.03 ± 0.01	0.03 ± 0.01	0.03 ± 0.01	0.03 ± 0.01
Ovary (Left)	0.03 ± 0.01	0.03 ± 0.01	0.03 ± 0.00	0.03 ± 0.01	0.03 ± 0.01
Uterus	0.77 ± 0.24	1.01 ± 0.30	1.07 ± 0.16	1.14 ± 0.20^a^	0.99 ± 0.40
Pups					
No of litters	9	8	10	10	9
Brain	4.62 ± 0.64	4.54 ± 0.51	4.71 ± 0.41	5.11 ± 0.45	4.90 ± 0.42
Lung	1.96 ± 0.13	2.08 ± 0.17	2.19 ± 0.22	2.22 ± 0.24	2.21 ± 0.23
Liver	4.05 ± 0.42	4.50 ± 0.44	4.44 ± 0.23	4.51 ± 0.54	4.36 ± 0.32
Kidneys	1.18 ± 0.16	1.11 ± 0.07	1.13 ± 0.06	1.25 ± 0.13	1.17 ± 0.05

Results are expressed as the mean ± SD. For offspring, Results were expressed as the mean of 6 pups in each litter ± SD. ^a^Significantly different from the control (*p* < 0.05), ^b^significantly different from low dose (*p* < 0.05)

### Ag distribution

In dams, Ag concentration increased numerically with Ag-NP dose in most tissues from 0.2 mg/kg, significantly so in spleen, kidney, uterus, plasma and erythrocytes (Table 3). Dose dependency was most outspoken in erythrocytes and plasma. The concentrations were generally even higher following treatment with AgNO_3_, especially for heart and plasma.

**Table 3:** Tissue content of Ag in dams (mean ± SD)

Silver (μg/g tissue)	Dose levels (mg/kg)				
	Control (*n* = 9)	0.2 Ag-NPs (*n* = 8)	2 Ag-NPs (*n* = 10)	20 Ag-NPs (*n* = 10)	20 AgNO_3_ (*n* = 9)
Spleen	0.09 ± 0.08	0.42 ± 0.33	0.35 ± 0.18^a^	0.84 ± 0.76	2.8 ± 1.43^a^
Kidney	0.33 ± 0.24	0.61 ± 0.34	1.37 ± 0.69	1.79 ± 0.72^a,b^	4.96 ± 3.22
Liver	0.46 ± 0.25	0.82 ± 0.57	0.83 ± 0.68	1.04 ± 0.49	1.27 ± 0.65
Uterus	0.34 ± 0.30	0.29 ± 0.17	0.58 ± 0.25	0.89 ± 0.10^a,b^	1.71 ± 1.00^a,b^
Heart	0.34 ± 0.28	0.31 ± 0.21	0.76 ± 0.68	0.88 ± 0.70	4.97 ± 0.78^a,b,c,d^
Lung	0.36 ± 0.16	0.31 ± 0.25	0.43 ± 0.39	0.66 ± 0.61	0.70 ± 0.34
Brain	0.17 ± 0.13	0.20 ± 0.09	0.24 ± 0.06	0.29 ± 0.09	0.42 ± 0.21^a,b,c^
Plasma	< LOD^*^	0.01 ± 0.01	0.08 ± 0.02^b^	0.17 ± 0.05^b,c^	0.67 ± 0.30^b,c,d^
Erythrocyte	< LOD	< LOD	0.01 ± 0.00	0.02 ± 0.01^c^	0.08 ± 0.02^c,d^
Milk	0.25 ± 0.21	0.33 ± 0.25	0.32 ± 0.11	0.66 ± 0.57	0.76 ± 0.45

^a^Significantly different from control (*p* < 0.05), ^b^Significantly different from low dose (*p* < 0.05), ^c^Significantly different from middle dose (*p* < 0.05), ^d^Significantly different from high dose (*p* < 0.05). *Limit of detection (1.5 ng/ml).Milk samples are composed from the average of three pups in each litter

In offspring, the Ag content was numerically higher in all treated groups compared to the controls. This included milk from the suckling pups. NP exposure elevated Ag levels statistically significantly in kidneys at all dose levels (*p* < 0.01, Table 4). Significant increments were not observed for any other organs or tissues. For milk it is possible that the continuous removal by the offspring prevented Ag from accumulating to any significant degree.

**Table 4:** Tissue content of Ag in pups (mean ± SD)

^*^Silver (μg/g tissue)	Dose levels (mg/kg)				
	Control ^**^(*n* = 9)	0.2 Ag-NPs (*n* = 8)	2 Ag-NPs (*n* = 10)	20 Ag-NPs (*n* = 10)	20 AgNO_3_ (*n* = 9)
Kidney	0.52 ± 0.37	3.06 ± 0.85^a^	3.19 ± 0.73^a^	3.05 ± 1.45^a^	1.44 ± 0.89
Lung	0.72 ± 0.53	2.84 ± 1.94	2.09 ± 1.35	3.07 ± 1.60	2.34 ± 0.67^a^
Liver	0.27 ± 0.14	0.58 ± 0.21	0.47 ± 0.52	0.88 ± 0.55	0.62 ± 0.58
Brain	0.13 ± 0.04	0.20 ± 0.13	0.26 ± 0.09	0.26 ± 0.06	0.26 ± 0.01
Plasma	< LOD^***^	< LOD	< LOD	0.01 ± 0.00	0.04 ± 0.01^b^
Erythrocyte	< LOD	< LOD	< LOD	0.02 ± 0.02	0.04 ± 0.03

*Results represent Ag content (μg/g). **n is composed from the average of three pups in each litter. ^a^Significantly different from control (*p* < 0.05). ^b^Significantly different from high dose (*p* < 0.05), ***Limit of detection (1.5 ng/ml)

AgNO_3_ elevated Ag levels significantly in offspring lungs (*p* < 0.05). The observation of higher Ag concentrations at organ levels following treatment with AgNO_3_ did however not repeat itself in offspring as offspring tissues levels of Ag were generally similar or lower if their dams had been exposed to AgNO_3_ rather than the Ag-NPs. Only for plasma did AgNO_3_ offspring present with statistically significantly higher concentration than in the corresponding Ag-NP group. Interestingly, AgNO_3_ pups did not present with elevated Ag level in kidneys compared to controls.

### ALT, AST and IL-6 levels

It was observed that the level of AST and IL-6 in dams was increased slightly in Ag treated groups compared to control. Similarly in pups, AST levels were increased in middle, high and AgNO_3_ groups as well as all Ag exposed groups in IL-6 levels compared to control. However, these differences were not statistically significant (*p* < 0.05). The highest values for AST and IL-6 were obtained for AgNO_3_ group in both dams and pups (Table 5). In addition, ALT levels were found to be same in all groups in either dams or pups.

**Table 5:** ALT, AST and IL-6 Levels in Dams and Pups

Groups	ALT (ng/mL)		AST (U/L)		IL-6 (ng/L)	
	Dams	Pups	Dams	Pups	Dams	Pups
Control (*n* = 9)	38.9 ± 18.3	16.2 ± 7.6	97.3 ± 15.8	59.9 ± 23.6	158.4 ± 12.1	94.2 ± 36.8
Low (*n* = 8)	36.4 ± 14.7	18.6 ± 11.7	105.7 ± 22.6	55.6 ± 10.5	167.9 ± 17.4	108.6 ± 16.6
Middle (*n* = 10)	42.3 ± 13.3	18.6 ± 4.5	113.8 ± 15.6	66.4 ± 12.9	173.3 ± 17.8	107.6 ± 36.1
High (*n* = 10)	34.9 ± 10.4	16.8 ± 9.2	113.1 ± 13.8	70.9 ± 26.9	172.4 ± 19.3	117.7 ± 42.7
AgNO_3_ (*n* = 10)	39.3 ± 15.1	18.3 ± 7.1	123.2 ± 24.3	84.5 ± 31.5	180.6 ± 30.6	133.5 ± 44.2

Blood samples were pooled from all pups in each litter. Results were expressed as the mean ± SD

### Oxidative stress parameters

**Fig. 2:**
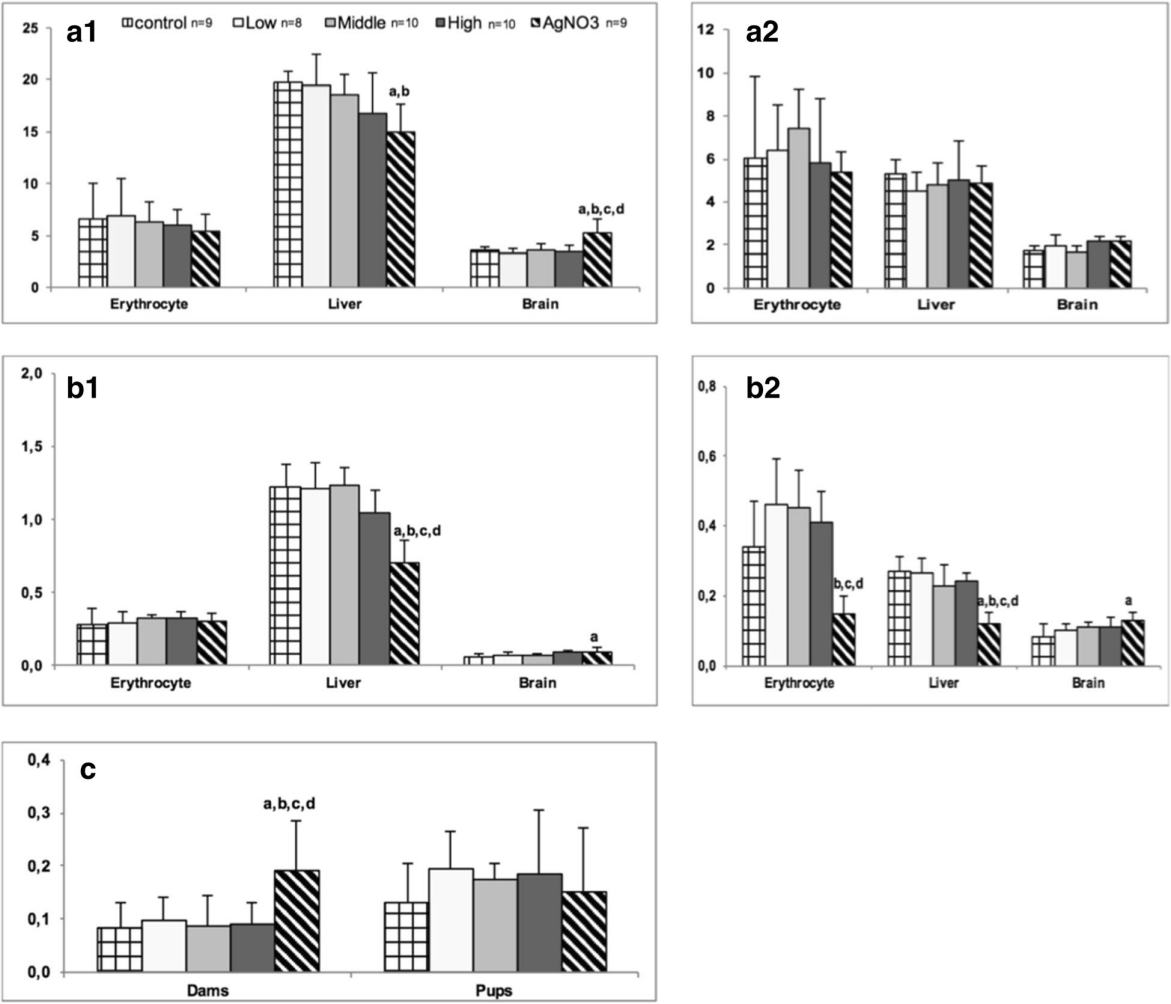
**a**1 SOD activities in dams (kU/g protein), **a**2 SOD activities in pups (kU/g protein), **b**1 GPx activities in dams (kU/g protein), **b**2 GPx activities in pups (kU/g protein). **c** Nitrite/Nitrate levels in plasma of dams and pups (nmol/mL). Results are expressed as mean ± SD for dams. ^a^significantly different from control (*p* < 0.05), ^b^significantly different from low dose (*p* < 0.05), ^c^significantly different from middle dose (*p* < 0.05), ^d^significantly different from high dose (*p* < 0.05)

Oxidative stress parameters are presented in Fig. 2. AgNO_3_ dams showed a significant decrease in liver SOD level compared to controls and low dose Ag-NPs (*p* < 0.05), whereas brain SOD level in AgNO_3_ dams were significantly increased compared to all other groups (*p* < 0.05). In offspring, no significant differences were observed.

GPx was significantly decreased in liver from AgNO_3_ exposed dams compared to all other groups (*p* < 0.001). In brain, GPx seemed to increase with dose, but only the AgNO_3_ group differed significantly from controls. In pups, the AgNO_3_ group exhibited significantly decreased GPx levels in liver (*p* < 0.01) compared to all other groups, and in erythrocytes compared to all other groups but controls (*p* < 0.01). GPx levels seemed to increase with dose in brain, but only offspring from the AgNO_3_ treated group differed significantly from controls (*p*˂0.05) (Fig. 2).

The plasma concentration NO_2_^-^/NO_3_^-^ almost doubled in AgNO_3_ exposed dams compared to all other groups (*p* < 0.01, Fig. 2).

Not significant changes were observed for CAT activities and MDA levels in dams or pups (data not shown).

### Histopathology

Histopathological findings for dams are summarized in Table 6. In dam liver, kidney and lung some minimal histological changes were observed with no such observations in controls.

**Table 6:** Histopathological findings for dams

Group				Control	Low	Middle	High	AgNo_3_
Number of animals (n)				5	5	5	5	5
				n	n	n	n	n
Liver	No microscopic findings			5/5	3/5	4/5	3/5	2/5
	Abnormality							
	Hyperplasia	Bile duct		0/5	0/5	0/5	0/5	0/5
	Vacuolation	hepatocellular	minimal	0/5	2/5	1/5	2/5	3/5
	Necrosis			0/5	0/5	0/5	0/5	0/5
	Hemorrhage			0/5	0/5	0/5	0/5	0/5
	Pigmentation			0/5	0/5	0/5	0/5	0/5
	Inflammation			0/5	0/5	0/5	0/5	0/5
Kidney	No microscopic findings			5/5	4/5	1/5	1/5	1/5
	Abnormality							
	Basophilia			0/5	0/5	0/5	0/5	0/5
	Inflammation		minimal	0/5	1/5	0/5	2/5	2/5
	Necrosis	tubular	minimal	0/5	1/5	4/5	3/5	3/5
Lung	No microscopic findings			5/5	2/5	3/5	1/5	1/5
	Abnormality							
	Inflammation		minimal	0/5	0/5	0/5	1/5	2/5
			mild	0/5	1/5	0/5	0/5	1/5
	Histiocytosis accumulation		minimal	0/5	2/5	1/5	3/5	1/5
	Mineralization			0/5	0/5	0/5	0/5	0/5
	Anthracosis			0/5	2/5	1/5	3/5	2/5
Spleen	No microscopic findings			5/5	5/5	4/5	3/5	2/5
	Abnormality							
	Infarct			0/5	0/5	0/5	0/5	0/5
	Inflammation			0/5	0/5	0/5	0/5	0/5
	Hyalinization	periarterial	minimal	0/5	0/5	1/5	2/5	3/5
Brain	No microscopic findings			5/5	1/5	2/5	1/5	1/5
	No of dams with neuronal loss event			0/5	4/5	3/5	4/5	4/5

**Fig. 3:**
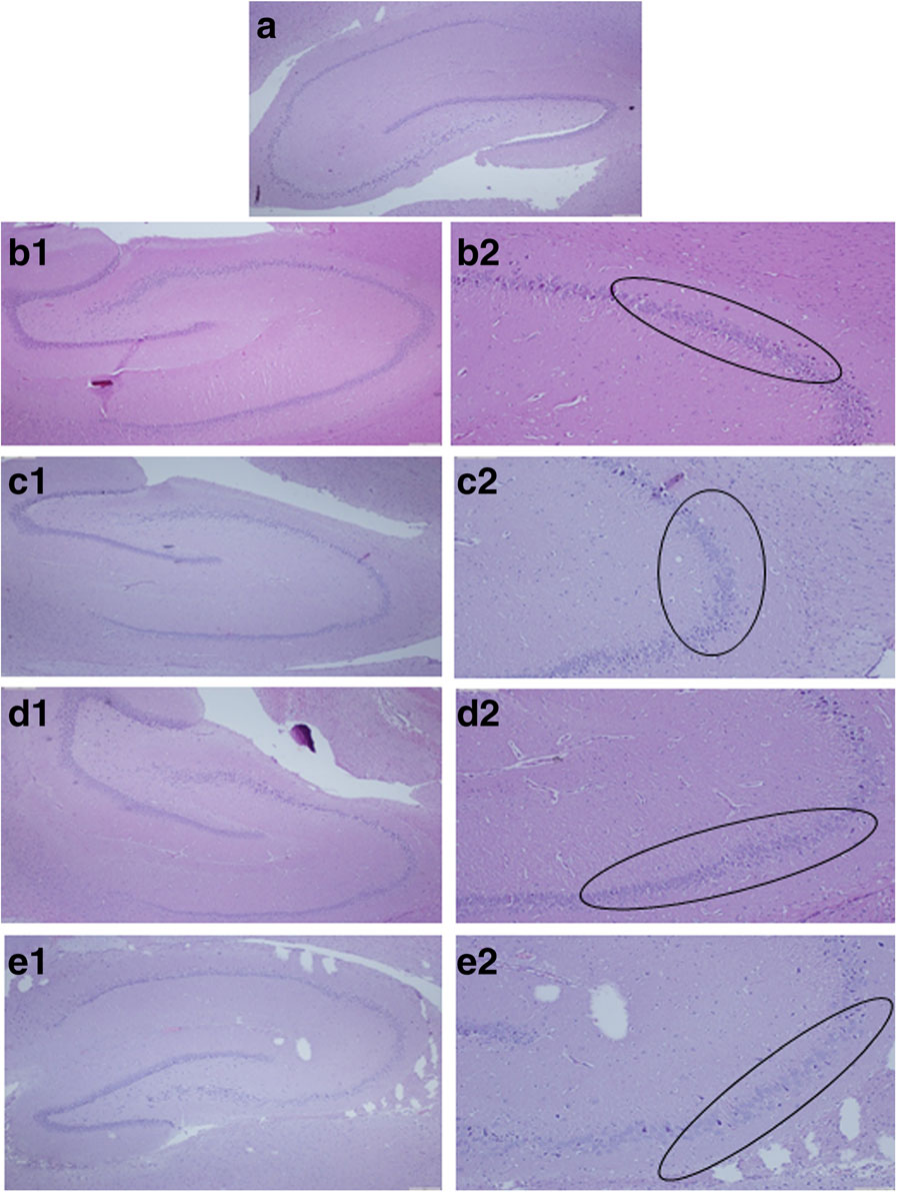
H&E, hippocampus of dams, CA1, CA2, CA3 and CA4 sectors. a) control (x40), b1) mild pyramidal neuronal loss in low dose Ag-NPs group (x40), b2) mild pyramidal neuronal loss in low dose Ag-NPs group (x100), c1) moderate pyramidal neuronal loss in middle dose Ag-NPs group (x40), c2) moderate pyramidal neuronal loss in middle dose Ag-NPs group (x100), d1) severe pyramidal neuronal loss in high dose Ag-NPs group (x40), d2) severe pyramidal neuronal loss in high dose Ag-NPs group (x100), e1) moderate pyramidal neuronal loss in AgNO_3_ group (x40), e2) moderate pyramidal neuronal loss in AgNO_3_ group (x100)

**Fig. 4:**
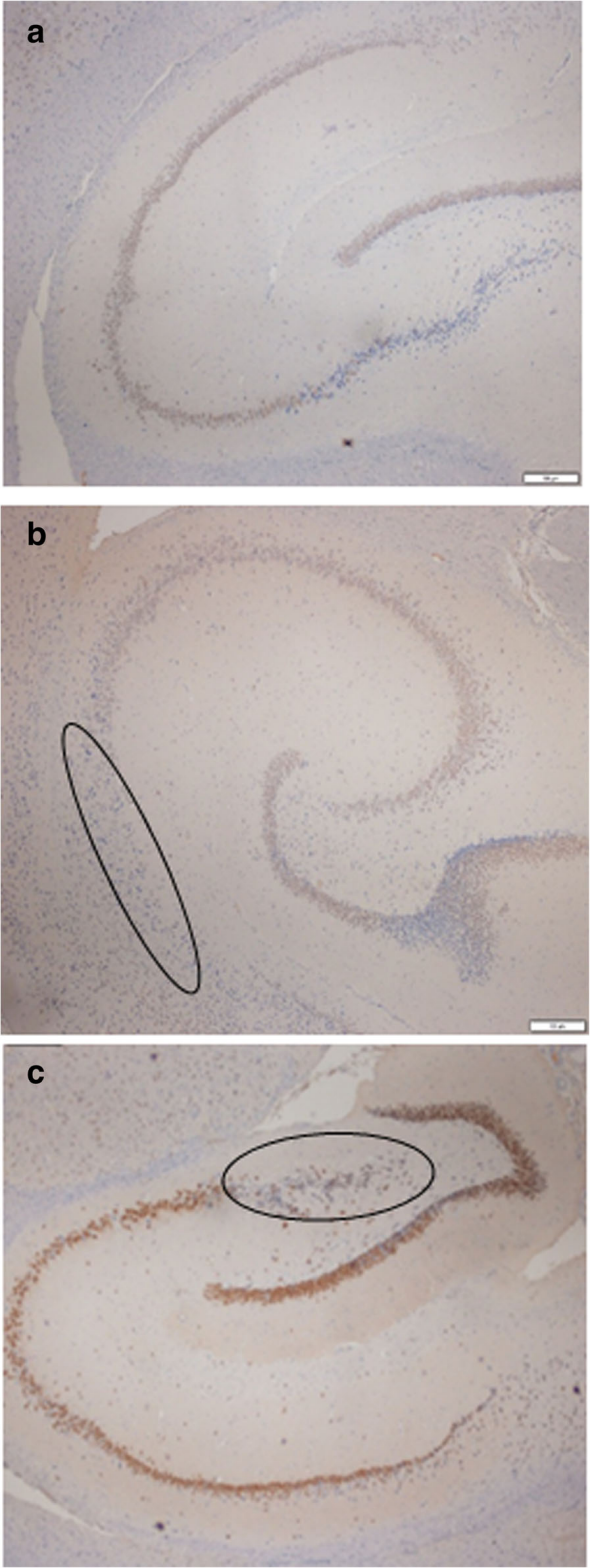
Neuronal specificity of nuclear staining by mAb in a section of hippocampus. **a** control, **b** mild-moderate pyramidal neuronal loss in CA1 sector edge neurons of high dose Ag-NPs group (x40), **c** mild-moderate pyramidal neuronal loss in in CA1 sector edge neurons of AgNO_3_ group (x40)

**Fig. 5:**
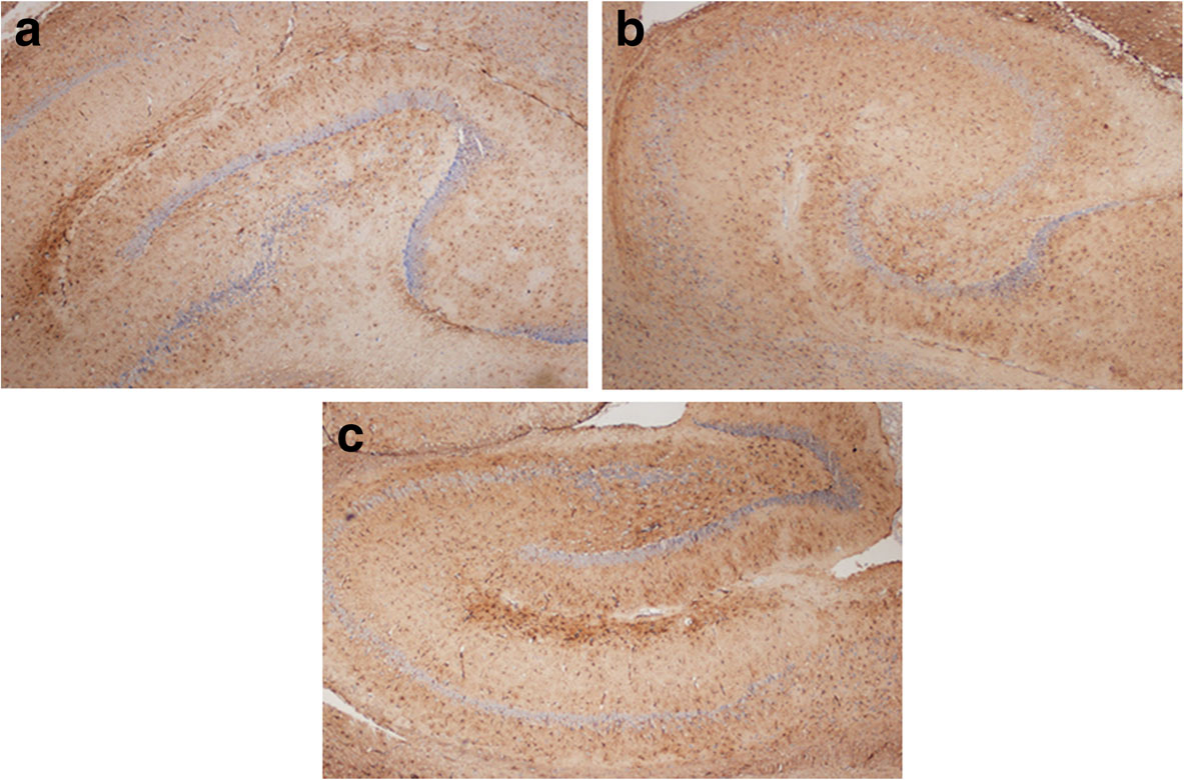
Differences in GFAP immunoreactivity between controls and Ag exposed groups. **a** control **b**) increased glial tissue; gliosis in high dose Ag-NPs group when compared to control section could be seen. **c** increased glial tissue; gliosis in AgNO_3_ group when compared to control section could be seen

In brains from exposed dams a high incidence of mild to moderate hippocampal pyramidal neuronal loss and mild gliosis were revealed (Figs. 3, 4 and 5). This event was observed in 4/5, 3/5, 4/5 and 4/5 of the low, middle, and high Ag-NPs dose groups and the AgNO_3_ dams, respectively (Table 6). Neuronal loss and mild gliosis (hippocampal sclerosis) was further classified according to the new International League against Epilepsy (ILAE) consensus (ILAE type 1: classical hippocampal sclerosis, ILAE type 2: CA1 sector sclerosis, ILAE type 3: CA4 sector sclerosis. Type 1 refers always to severe neuronal cell loss and gliosis predominantly in CA1 and CA4 sectors, compared to CA1 predominant neuronal cell loss and gliosis (type 2), CA4 predominant neuronal cell loss and gliosis (type 3) [26]. In our study, mild to moderate neuronal cell loss and gliosis event in animals exposed to Ag in nanoparticulate or ionic forms was observed predominantly in CA1 sector, which can be categorized as a type 2 hippocampal sclerosis according to the ILAE classification. Normal morphology of pyramidal neurons in all regions of the hippocampus was observed in dams from the control group.

Histopathological examination of brain, heart, liver, kidney and lung tissues of the offspring did not reveal changes related to treatment.

## Discussion

The increasing application of silver in both ionic and nanoparticle forms has led to concerns regarding the potential health impacts of human exposure to this material.

Up to 1000 mg/kg/day were utilized in a 28-day study on (adult) oral toxicity of Ag-NPs, and a NOAEL value of 30 mg/kg was suggested by the authors [27]. Another study indicated NOAEL values of less than 100 mg/kg/day for dams and 1000 mg/kg/day for embryo-fetal development [3]. The present study, attempted to identify the potential adverse effects of Ag in the form of Ag-NPs and ionic Ag at exposure levels below the NOAEL values indicated in previous studies. The daily human dietary silver intake has been estimated to range from 0.4 to 27 μg/day [28]. Although it is difficult to predict human exposure level to Ag-NPs, some reports estimate level of < 100 μg/day as a realistic dose for Ag-NPs toxicity studies [29]. Although the applied dose levels are in excess of the estimated human intake, these doses are not exaggerated considering the short period of exposure (14-days) and the long retention of Ag once it has been taken up in e.g. the brain [30].

Since the Ag-NPs were prepared through the reduction of Ag^+^ ions, it is possible that some unreduced Ag^+^ ions could have been present in the final suspension. In order to eliminate interference from free Ag^+^ ions in the prepared dosing suspensions, approximately 3 times the required trisodium citrate were utilized during the syntheses process of Ag-NPs. According to the chemical reaction of the synthesis process, 34 mg of trisodium citrate were required to convert the total amount of utilized Ag (57 mg) to Ag-NPs. Nevertheless, we utilized 100 mg of trisodium citrate to ensure all Ag ions were converted to the nanoparticles form of Ag. Following the synthesis process, the NPs were washed thrice by precipitation and re-suspension in water to ensure removal of citrate ions. Dissolution of Ag-NPs after the reductions have taken place presents another possibility for introduction of Ag + ions to the dosing suspension. Therefore, the NPs were produced fresh thrice weekly. Consequently, the influence of Ag and citrate ions in the prepared suspension would be negligible at the time of treatment.

### Findings on pregnancy and fetuses

No treatment related effects were found for the gestational parameters of pregnancy length, number of implantations, resorptions, birth weight and litter size. The findings on Ag-NPs support the previous reports that demonstrate no signs of developmental toxicity for up to 1000 mg/kg Ag-NPs/day [3, 12]. Maternal weight gain was however significantly lower in dams receiving AgNO_3_ compared to all other groups. This indicates that the ionic form may induce a higher degree of maternal toxicity compared to the NP form.

### Ag distribution

In the Ag-NPs treated groups the maternal blood Ag content increased significantly in a dose dependent manner. The high Ag concentration in plasma relative to erythrocytes corroborates a previous report indicating a high degree of serum protein binding for Ag [31]. The maternal blood Ag level in the AgNO_3_ group was significantly higher than that of the NP group at a similar Ag dose level, indicating a higher gastrointestinal absorption rate of Ag as AgNO_3_ compared the NP form. This is consistent with the findings in a 28-day study in which rats were exposed orally to 14 nm Ag-NPs or Ag acetate [32]. Lower tissue Ag levels and higher fecal excretion were observed for Ag-NPs compared to Ag as acetate. The authors suggested that enhanced binding of Ag-NPs to non-digestible food components in the intestines because of their large active surfaces, could have increased excretion and thereby decreased absorption.

The much lower Ag content in maternal blood compared to tissues indicates that Ag was sequestered from blood to organs. At the highest dose level of Ag-NPs, maternal spleen and kidney presented with the highest Ag accumulation, followed by liver, uterus and heart compared to controls. Previous studies demonstrate that liver and kidney are the main target organs for silver distribution upon oral exposure to 60 nm Ag-NPs (spleen not measured) [27, 33]. Nanoparticles have however also been reported to be absorbed via the lymphatic system. Accordingly, the lower distribution of Ag to liver compared to spleen and kidney may be explained by NP binding to long chain fatty acids taken up via the lymphatic system as this would bypass the first-pass effect of the liver. Overall, the relative absorption of NPs probably depends on factors such as particle size, surface charge, hydrophobicity and the presence or absence of surface ligands [34].

Accumulation of Ag in offspring confirms that Ag is able to cross the placenta. The kidney seem to be the main organ of fetal accumulation as it exhibited a 5.8-fold higher level of Ag compared to control offspring kidneys at the highest dose level of Ag-NPs, followed by lung, liver and brain. This distribution pattern compares well to the results from Lee et al. where 250 mg/kg/day citrate-coated Ag-NPs (7.9 nm) were administered orally to pregnant rats. Offspring kidney was the primary organ for Ag accumulation. Of note, kidney Ag levels were only twice as high as in our study even if they used a much higher dose and smaller particles [4]. It indicates, together with our observation of approximately similar fetal Ag contents at all Ag-NPs exposure levels, that the placenta might limit the transfer of ionic Ag and/or Ag-NPs from the maternal to the fetal compartment.

In dams, administration of AgNO_3_ lead to higher tissue contents of Ag than did administration of Ag-NPs. The similar or even lower organ contents of Ag in offspring of the AgNO_3_ group compared to the Ag-NPs groups was therefore surprising. It could be speculated if this observation should be explained by differential translocation of Ag in nanoparticulate and ionic forms across the placental barrier. However, offspring blood Ag levels was elevated in the AgNO_3_ group compared to the Ag-NPs group.

To our knowledge, this is the only study comparing the distribution pattern of Ag in rat offspring organs from the NPs compared to a soluble salt. The difference in fetal distribution patterns makes it unlikely that the Ag-NPs dissolved immediately into silver ions. A fraction of the administered Ag may therefore have reached the fetuses in particulate form, providing some explanation of the higher Ag levels in Ag-NP compared to AgNO_3_ pups in some organs. Once in the fetal tissue, particles could continue release of Ag. This is well-described as the Trojan-horse mechanism, by which the particulate form facilitates uptake and increases the cellular bioavailability to silver at a later stage [20, 35].

### ALT, AST and IL-6 levels

The results of the biochemical analysis of two biomarkers of hepatotoxicity, ALT and AST, in plasma showed no significant differences between groups, which may be determinative of no acute hepatotoxicity indication. However, in spite of not significant increment in ALT and AST levels, some minimal adverse responses were still observed in histopathological analysis of liver. Our findings on the ALT and AST levels are compatible with the report by van der Zande et al. where no changes in ALT and AST levels were found after 28-day oral exposure to Ag-NPs (90 mg/kg/day) and AgNO_3_ (9 mg/kg/day) in rats [5].

Toxicity of NPs was manifested by inflammation resulting from oxidative stress [2, 36]. In previous study, it was reported that when mice were repeatedly exposed for 28 days by oral administration with 0.25, 0.50 and 1.00 mg/kg dose of Ag-NPs, these NPs induce cytokine level of blood in a dose dependent manner [36]. In contrast, Daniel et al. reported that intra-peritoneal injection of Ag-NPs in to mice showed no immune response. However, increase in CAT activity and decrease in GSH content following administration of Ag-NPs in that study, confirming the oxidative stress capacity of Ag-NPs [37]. In current study, no statistically significant changes were found in IL-6 levels of plasma between groups either in dams or pups up to daily dose of 20 mg/kg. However, IL-6 was slightly increased in both Ag-NPs and AgNO_3_ treated dams as well as related offspring versus to control. AgNO_3_ group also showed the most induction of IL-6 among Ag treated groups. This induction may be in accordance with our findings on oxidative stress parameters. Subsequently, this induction may also be in accordance with findings of histopathological examinations which demonstrated higher incidence of inflammation in the form of lymphocyte influx in both Ag-NPs and AgNO_3_ treated dams compared to the control group.

### Oxidative stress

Administration of AgNO_3_ to pregnant rats decreased SOD activity in liver by ~25 % compared to control and low dose Ag-NPs dams. An almost parallel (albeit statistically insignificant) decrease was observed in erythrocytes (~20 %). Exposure to Ag-NPs has been associated with increased generation of ROS [38]. SOD catalyzes the dismutation of superoxide into oxygen and hydrogen peroxide to protect the cell [39]. The observed reduction in SOD activity could therefore be due to increased production of H_2_O_2_ in liver. The decreased GPx activity in liver may result from the involvement of this enzyme in the scavenging of peroxides generated during Ag biotransformation [40]. GPx activity was however also decreased in pup liver and erythrocytes, but without corresponding increases in SOD activity. Silver ions have also been shown to substitute copper on ceruloplasmin which may dramatically decrease the content of copper in plasma. Since SOD is co-factored by copper, lack of copper may decrease the activity of SOD. Thus, feeding rats on a diet with silver salts (50 mg AgCl/day) for three weeks reduced plasma levels of Cu (by 30 %) and interfered with SOD activity, probably through inhibition of ceruloplasmin [18]. Only a slight dose dependent decrease in SOD was observed in erythrocytes and liver homogenate of Ag-NPs exposed dams,

In brain, AgNO_3_ significantly increased SOD levels compared to all other groups in dams and in pups. The reverse trend of SOD in liver and brain may be owe to brain tissue being more susceptible to oxidative failure of the total antioxidant defense during pregnancy [41]. The elevated activity of brain SOD may therefore be due to an adaptive response to free radicals generated due to Ag exposure, especially outspoken in AgNO_3_ exposed females where Ag content was almost doubled compared to Ag-NP exposed females. Increased activity of brain GPx in AgNO_3_ treated dams may be similarly explained an adaptive response.

Overall, our findings of decreased levels of SOD and GPx levels in dam liver and of increased NO_2_^-^/NO_3_^-^ levels in maternal plasma indicate that Ag may induce oxidative stress. Changes in pup GPx indicate that oxidative stress may also have been induced in fetal tissues. This was however apparent only when Ag was administrated in ionic form. It can be concluded that the oxidative response/damage of Ag-NPs reported in previous studies depends not only on the NPs, but also the amount of Ag ions released from the surface of the NPs.

### Histopathology

The high incidence of hippocampal neuronal cell loss observed in all Ag dose groups in combination with increased in Ag levels in brain are alarming. This event is even more disturbing in the light of previous studies which report long retention of Ag in brain tissue [5, 30]. Fortunately, no corresponding effect was observed in fetal brains. The change in offspring GPx activity does however indicate that the fetal brain is susceptible to maternal Ag exposure.

The hippocampus plays an important role in formation of memory as well as detection of novel events, places and stimuli [42]. Liu et al. investigated neurobehavioral effects of Ag-NPs following nasal administration to adult rats. Ag-NPs induced learning and memory deficits even at a dose level of 3 mg/kg. Histopathological assessment clearly showed edema and nuclear shrinking as well as neorobiosis in the hippocampal pyramidal neurons. This was further supported by the presence of superoxide anions and hydroxyl radicals in the hippocampus [12]. However, it should be noted that the nasal route of exposure is much closer to the brain than the gastrointestinal tract and that NPs have been suggested to be taken up the brain through the olfactory nerve [43]. Also, it is not clear whether the effects occurred due to the Ag-NPs or released Ag ions since a soluble Ag control were not included in the study [8].

The present study shows that oral administration of Ag, in both NP and ionic forms may generate hippocampal neuronal damage. Only Ag in the ionic form significantly increased GPx activity in adult brain, but GPx activity was increased numerically and dose dependently also after the administration of Ag-NPs. Possibly, oxidative stress may lead to hippocampal sclerosis even at very low levels of exposure, since similar incidences were observed in all treated dams in this study. Of concern, AgNO_3_ exposure also increased GPx levels in offspring brain indicating induction of oxidative stress. As in dams, GPx activity apparently increased dose dependently also for Ag-NPs, although it was non-significantly. However, the histopathological examinations of offspring tissues revealed no morphological changes in this study, but volumetric measurement of developing rat hippocampi has previously showed that Ag decreased the total volume of hippocampal pyramidal cells [44]. Wu et al. investigated the effect of prenatal exposure to PVP coated and uncoated Ag-NPs on spatial cognition and hippocampal neurodevelopment in rats. Results revealed that administration of uncoated Ag-NPs to mothers during pregnancy impairs spatial learning and memory ability in rat offspring. Coating with PVP protected offspring from the toxic effects of Ag-NPs in that study [45]. Ghaderi et al. reported that subcutaneous injection of Ag-NPs during pregnancy could induce adverse consequences on neurobehavioral development of offspring because of its damage on spatial cognition [17].

## Conclusion

The present study shows that administration of Ag-NPs during gestation did not cause developmental toxicity at dose levels of up to 20 mg/kg/day as judged by traditional pregnancy and developmental parameters. It is however shown that the prenatal exposure to Ag in both ionic and nanoparticle forms increase the levels of Ag in offspring tissues. The underlying kinetics differs between the ﻿two﻿ forms of Ag. The ionic Ag was associated with a higher degree of toxicity. The Ag in both nanoparticle and ionic forms induced oxidative stress in dams and pups, again with the ionic form being more potent. Observation of hippocampal sclerosis even at the lowest dose level of 0.2 mg/kg/day is alarming and the NOAEL values reported in previous studies may be need adjustment if our findings are corroborated. Finally, the observation of oxidative stress in offspring brain tissue is truly worrying. As the developing brain is considered more vulnerable to toxic insults than the adult nervous system [46] further studies of the developmental neurotoxicity of Ag in ionic as well as nanoparticle form are called for, preferably with inclusion of histological as well as long-term behavioral measures.
